# Brain perfusion in fibromyalgia patients and its differences between responders and poor responders to gabapentin

**DOI:** 10.1186/ar2980

**Published:** 2010-04-07

**Authors:** Chie Usui, Kotaro Hatta, Nagafumi Doi, Atsushi Nakanishi, Hiroyuki Nakamura, Kusuki Nishioka, Heii Arai

**Affiliations:** 1 Department of Psychiatry, Juntendo University School of Medicine, 2-1-1 Hongo, Bunkyo-Ku, Tokyo 113-8421, Japan; 2 Ibaraki Prefectural Tomobe Hospital, 654 asahi-cho, kasama-city, Ibaraki 309-1717, Japan; 3 Department of Radiology, Juntendo University School of Medicine, 2-1-1 Hongo, Bunkyo-Ku, Tokyo 113-8421, Japan; 4 Department of Environmental and Preventive Medicine, Graduate School of Medical Science, Kanazawa University, kakuma-cho, Kanazawa-city, Kanazawa 920-1192, Japan; 5 Institute of Innovative Medical Science and Education, Tokyo Medical University, 6-1-1 Shinjyuku, Shinjyuku-ku, Tokyo 160-8402, Japan

## Abstract

**Introduction:**

The aim of the present study was to determine the brain areas associated with fibromyalgia, and whether pretreatment regional cerebral blood flow (rCBF) can predict response to gabapentin treatment.

**Methods:**

A total of 29 women with fibromyalgia and 10 healthy women (without pain) matched for age were finally enrolled in the study. Technetium-99m ethyl cysteinate dimer single photon emission computed tomography (^99m^Tc-ECD SPECT) was performed in the fibromyalgia patients and controls. A voxel-by-voxel group analysis was performed using Statistic Parametric Mapping 5 (SPM5). After treatment with gabapentin, 16 patients were considered 'responders', with decrease in pain of greater than 50% as evaluated by visual analogue scale (VAS). The remaining 13 patients were considered 'poor responders'.

**Results:**

We observed rCBF abnormalities, compared to control subjects, in fibromyalgia including hypoperfusion in the left culmen and hyperperfusion in the right precentral gyrus, right posterior cingulate, right superior occipital gyrus, right cuneus, left inferior parietal lobule, right middle temporal gyrus, left postcentral gyrus, and left superior parietal lobule. Compared to responders, poor responders exhibited hyperperfusion in the right middle temporal gyrus, left middle frontal gyrus, left superior frontal gyrus, right postcentral gyrus, right precuneus, right cingulate, left middle occipital gyrus, and left declive. The right middle temporal gyrus, left superior frontal gyrus, right precuneus, left middle occipital gyrus, and left declive exhibited high positive likelihood ratios.

**Conclusions:**

The present study revealed brain regions with significant hyperperfusion associated with the default-mode network, in addition to abnormalities in the sensory dimension of pain processing and affective-attentional areas in fibromyalgia patients. Furthermore, hyperperfusion in these areas was strongly predictive of poor response to gabapentin.

## Introduction

Fibromyalgia (FM) is characterized by widespread musculoskeletal chronic pain, fatigue, poor sleep, frequent psychological difficulties, and multiple tender points (TPs) on physical examination [1]. Although neither the etiology nor the pathogenesis of this condition is fully understood, FM appears to be a disorder of the central nervous system (CNS) and a type of central sensitivity syndrome [2]; thus, brain imaging studies of patients with FM have been performed. Mountz and colleagues [3] found hypoperfusion in the bilateral thalamus and bilateral caudate nucleus using technetium-99m hexamethylpropylene amine oxime (^99m^Tc-HMPAO) single-photon emission computed tomography (SPECT). Kwiatek and colleagues [4] also found hypoperfusion in the right thalamus and in a region near the right lentiform nucleus in the caudate nucleus as well as inferior pontine tegmentum using ^99m^Tc-HMPAO SPECT. Guedj and colleagues [5,6] found hyperperfusion in the somatosensory cortex and hypoperfusion in the bilateral medial frontal, bilateral anterior cingulate, bilateral posterior cingulate, and cerebellar cortices using ^99m^Tc-ethyl cysteinate dimer (ECD) SPECT. Thus, although several SPECT studies revealed abnormal regional cerebral blood flow (rCBF) in patients with FM, the findings have not been consistent with each other.

Gabapentin is known to relieve neuropathic pain as well as antidepressants in the treatment of FM [7]. Recently, the effectiveness of gabapentin was demonstrated in a randomized, double-blind, placebo-controlled clinical trial [7]. However, differences between responders to gabapentin and non-responders were unclear. The aim of the present study was to determine the brain areas associated with FM using SPECT and whether pretreatment rCBF can predict response to gabapentin.

## Materials and methods

### Subjects

A total of 29 women with FM (45.1 ± 12.6 years of age, range 22 to 67 years) who met the American College of Rheumatology criteria [1], did not suffer from major depression according to DSM-IV (*Diagnostic and Statistical Manual of Mental Disorders, Fourth Edition*), and visited the Juntendo University Hospital as outpatients between July 2007 and April 2008 were enrolled in the study. None of the patients had any other significant medical illnesses such as stroke or malignancy with suspicion of metastasis on computed tomography scan or brain magnetic resonance imaging. Laboratory examination, including complete blood count, C-reactive protein, rheumatoid factor, anti-nuclear antibody, C3 and C4 levels, and anti-ds-DNA tests, were performed to exclude any underlying disease. All patients were CNS drug-naive. For comparison of imaging findings, 10 healthy women without pain matched for age were also included as a control group (44.5 ± 7.6 years). This study was approved by the Ethics Committee of the Juntendo University School of Medicine. All subjects provided informed consent in accordance with institutional guidelines.

### Gabapentin therapy

Gabapentin was titrated in the following manner: 300 mg/day for the first week, 600 mg/day for the second week, 1,200 mg/day for the third week, 1,800 mg/day for the fourth week, and 2,400 mg/day for the fifth week. If a patient could not tolerate 2,400 mg/day, the dosage was reduced to a minimum of 1,200 mg/day. No additional medication or new therapy was used during the course of this study.

### SPECT protocol and analysis

Patients were injected with 600 MBq of ^99m^Tc-ECD. Radionuclide angiography was performed immediately after intravenous bolus injection of 3 mL of ^99m^Tc-ECD. A three-headed rotating gamma camera (Toshiba GCA-9300A/DI; Toshiba Corporation, Tokyo, Japan) was used for data acquisition, and a medical image processor (GMS5500; Toshiba Corporation) was employed for image processing. The energy window for acquisition was set at 140 keV with a width of 20%. The gamma camera was rotated continuously for 16 minutes, and SPECT data were arranged into 90 projections over 360°.

Acquired images were reconstructed in a 128 × 128 matrix with a pixel size of 1.72 mm using a ramp filter after being processed with a Butterworth filter (order 8, 0.13 to 0.15 cycles per pixel). Attenuation was corrected using Chang's method (attenuation coefficient μ = 0.09 cm^2^/g). The projection data were reformatted to construct transaxial images parallel to the orbitomeatal line. The pixel size and slice thickness were 3.44 mm^2 ^and 3.44 mm, respectively. A voxel-by-voxel group study was then performed using Statistic Parametric Mapping 5 (SPM5) (Welcome Department of Cognitive Neurology, University College, London, UK, running on MATLAB version R2007a, The MathWorks, Inc., Natick, MA, USA). Images were initially converted from ACR-NEMA1 to analyze format using MRIcro and transferred to SPM5. The data were then standardized with the Montreal Neurological Institute (MNI) atlas using a 12-paramter affine transformation, followed by non-linear transformations and a trilinear interpolation. Dimensions of resulting voxels were 2 × 2 × 2 mm. Standardized data were smoothed by a Gaussian filter (FWHM [full width at half maximum] 12 mm). The FM and control groups were compared using the 'compare-populations one scan/subject' routine, which carries out a fixed-effects simple *t *test for each voxel. Global normalization was performed using proportional scaling. The SPM t maps were initially obtained at a height threshold of *P *< 0.001, and then an extent threshold of 50 voxels was applied to obtain a statistical threshold corrected for multiple comparisons for the cluster (*P *< 0.05). MNI coordinates were finally converted to Talairach coordinates using the Pick Atlas [8]. Using SPM5 the plot of adjusted normalized rCBF in the voxel with the maximum value of each eligible cluster was obtained, and the predictive values for discrimination of the two subgroups of FM patients according to gabapentin response were calculated. Normalized perfusion values of significant clusters were then extracted, and MNI coordinates were finally converted to Talairach coordinates using the Talairach Daemon database. The Mann-Whitney *U *test was used to compare the adjusted normalized rCBF subgroups of patients and controls. To specify the relevance of visual analogue scale (VAS) pain score, TP score, and duration of pain to rCBF, we performed a multiple analysis, using VAS score, TP score, and duration of pain as predictors, which tested the relationship between a predictor and an outcome (*P *< 0.05, corrected for multiple comparison) in FM patients.

### Pain assessment

Patients were considered responders when they exhibited a decrease in VAS for pain of greater than 50% after treatment. Other patients were considered poor responders. Differences between the responder group and the poor responder group in demography and clinical characteristics were calculated with the unpaired *t *test. If data were not sampled from Gaussian distributions, a non-parametric test (Mann-Whitney *U *test) was used. All statistical tests were two-tailed. Statistical significance was set at *P *< 0.05.

## Results

### Patient disposition

During the study period, 35 patients with FM were registered, but due to adverse events, 6 patients discontinued the study during the 12-week therapy phase. All 6 patients complained of headache and dizziness. A total of 29 patients with FM completed the therapy phase; 16 of them were considered 'responders', whereas 13 of them were considered 'poor responders'. In the responder group, 11 of 16 patients could tolerate the full dose of gabapentin (2,400 mg/day), whereas 5 patients could not: among these 5 patients, 2 could tolerate 1,200 mg/day, 2 could tolerate 1,800 mg/day, and 1 could tolerate 2,100 mg/day. In the poor responder group, 9 of 11 patients could tolerate the full dose, whereas 2 patients could not (1,200 mg/day). There was no significant difference in the proportion of patients who could tolerate the full dose of gabapentin between the responder and poor responder groups (69% versus 82%, *P *= 0.66).

### Comparison of regional cerebral blood flow between the fibromyalgia and control groups

**Figure 1:**
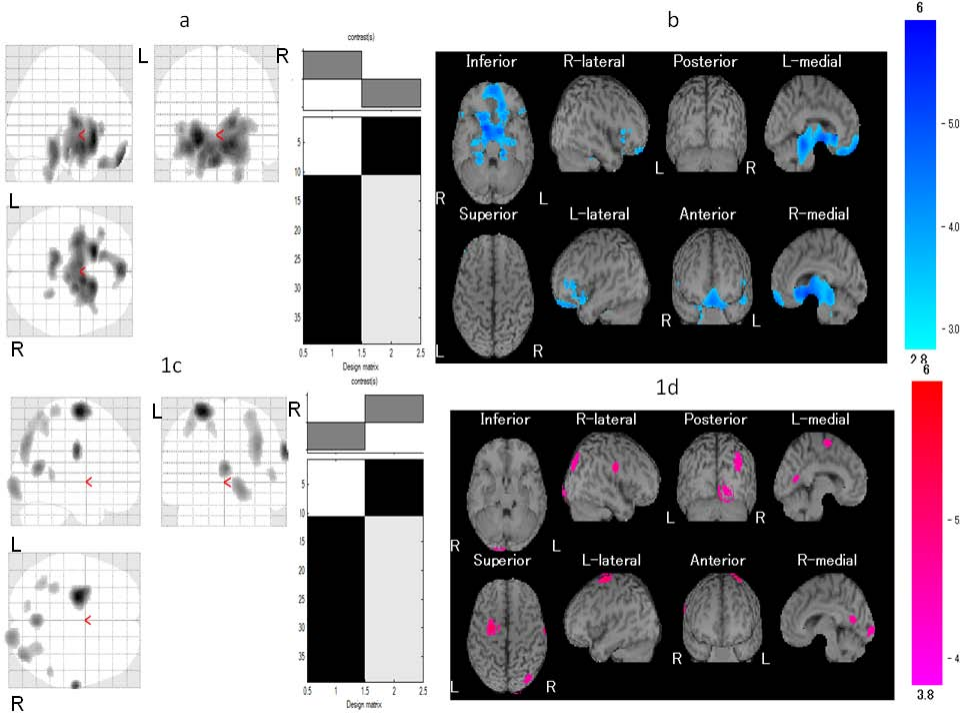
**Comparison of regional cerebral blood flow (rCBF) between patients with fibromyalgia (FM) and age-matched healthy controls**. Maximum intensity projections of Statistic Parametric Mapping 2 (SPM2) results from comparison of rCBF between patients with FM and age-matched healthy controls are shown. The FM patient group exhibited **(a, b) **significant hypoperfusion in the left culmen and **(c, d) **significant hyperperfusion in the right precentral gyrus, right posterior cingulate, right superior occipital gyrus, right cuneus, left inferior parietal lobule, right middle temporal gyrus, left postcentral gyrus, and left superior parietal lobule. Height threshold is less than 0.001, corrected for multiple comparison.

The FM patient group exhibited significant hypoperfusion in the left culmen (Table 1 and Figure 1a, b) and hyperperfusion in the right precentral gyrus, right posterior cingulate, right superior occipital gyrus, right cuneus, left inferior parietal lobule, right middle temporal gyrus, left postcentral gyrus, and left superior parietal lobule (Table 1 and Figure 1c, d). However, we found no significant correlations between rCBF and cognitive indicators of pain such as VAS score, TP score, and duration of pain.

**Table 1:** Regions of significant hyperperfusion and hypoperfusion in the fibromyalgia group

	κ	Z score	x, mm	y, mm	z, mm	Localization
Hyperperfusion	134	4.55	66	-10	30	Right precentral gyrus
	262	4.16	2	-62	14	Right posterior cingulate
	824	3.98	36	-82	32	Right superior occipital gyrus
	429	3.95	18	-96	-6	Right cuneus
	220	3.57	50	-38	52	Left inferior parietal lobule
	55	3.54	52	-46	6	Right middle temporal gyrus
	113	3.52	-30	-42	68	Left postcentral gyrus
		3.74	-14	-74	56	Left superior parietal lobule
	709	4.66	-2	56	-22	Left superior frontal gyrus
Hypoperfusion	1,111	4.38	-12	-32	-18	Left culmen

Results are listed by clusters. K value, Z score, Talairach coordinates of peak voxel, and anatomic localization are provided for each cluster.

### Comparison of regional cerebral blood flow among the responder, poor responder, and control groups

**Figure 2:**
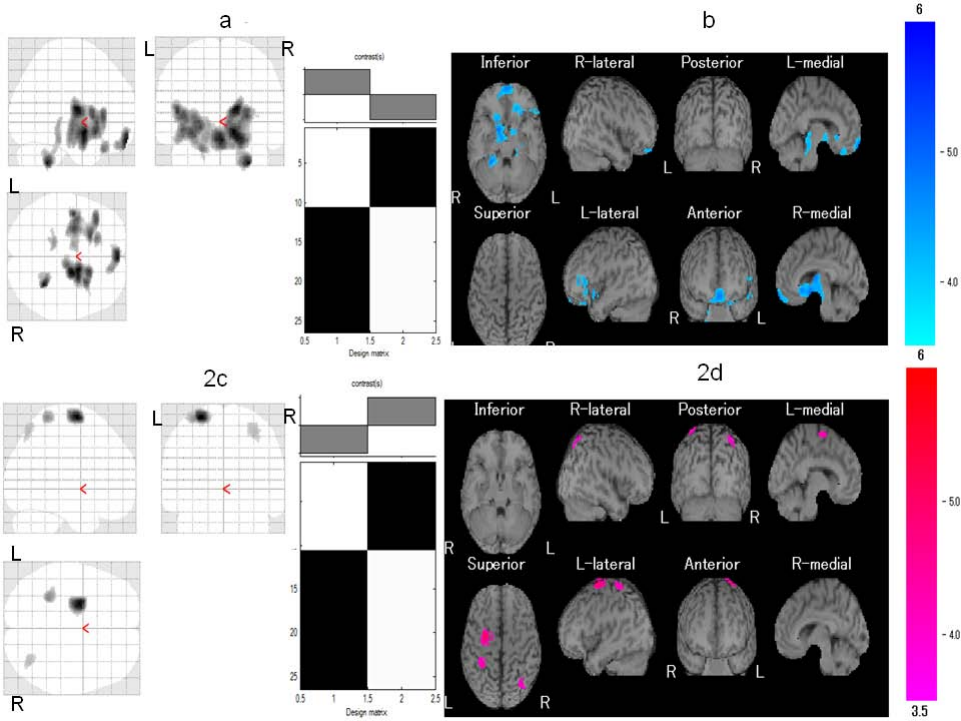
**Comparison of regional cerebral blood flow (rCBF) between the responder group and age-matched healthy controls**. Maximum intensity projections of Statistic Parametric Mapping 2 (SPM2) results from comparison of rCBF between the responder and age-matched healthy controls are shown. The responder group exhibited **(a, b) **significant hypoperfusion in the right medial frontal gyrus, left insula, left inferior frontal gyrus, and left culmen in the cerebellum and **(c, d) **significant hyperperfusion in the left superior frontal gyrus and the left postcentral gyrus. Height threshold is less than 0.001, corrected for multiple comparison.

**Figure 3:**
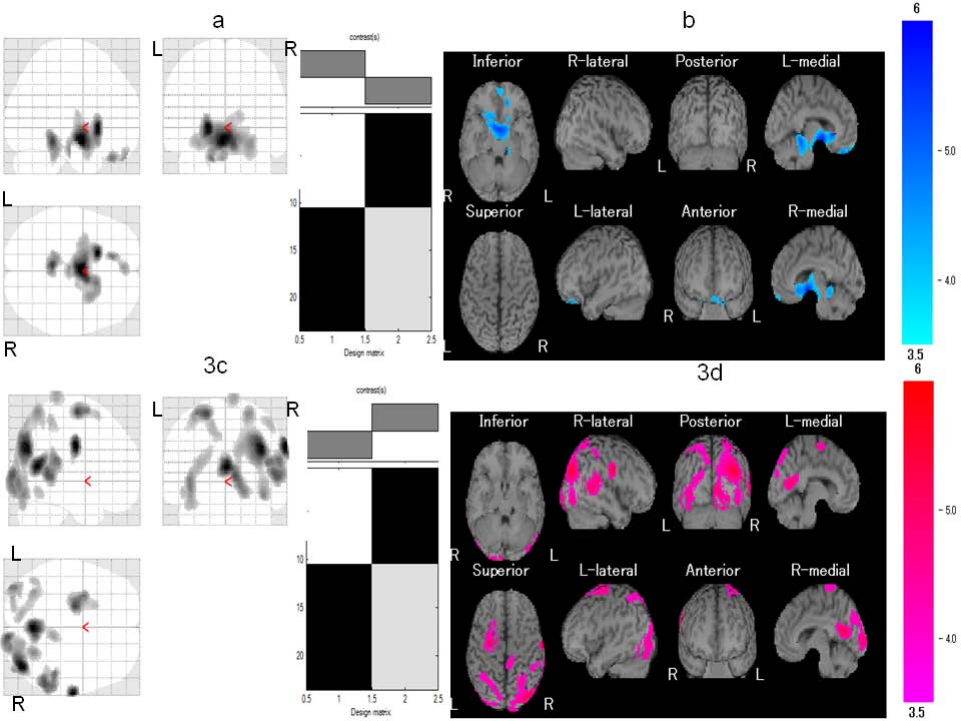
**Comparison of regional cerebral blood flow (rCBF) between the poor responder group and age-matched healthy controls**. Maximum intensity projections of Statistic Parametric Mapping 2 (SPM2) results from comparison of rCBF between the poor responder and age-matched healthy controls are shown. The poor responder group exhibited **(a, b) **significant hypoperfusion the left orbital gyrus and **(c, d) **significant hyperperfusion in the right precentral gyrus, right posterior cingulate, right precuneus, left middle frontal gyrus, right superior temporal gyrus, left middle occipital gyrus, right postcentral gyrus, and right inferior parietal lobule. Height threshold is less than 0.001, corrected for multiple comparison.

Demographic features and clinical features of the study participants are shown in Table 2. The responder and poor responder groups did not differ significantly in mean age, pain duration, VAS for pain, or number of TPs. Compared with the control group, the responder group exhibited significant hypoperfusion in the right medial frontal gyrus, left insula, left inferior frontal gyrus, and left culmen in the cerebellum (Table 3 and Figure 2a, b) as well as hyperperfusion in the left superior frontal gyrus and the left postcentral gyrus (Table 3 and Figure 2c, d). On the other hand, compared with the control group, the poor responders exhibited significant hypoperfusion in the left orbital gyrus (Table 4 and Figure 3a, b) and hyperperfusion in the right precentral gyrus, right posterior cingulate, right precuneus, left middle frontal gyrus, right superior temporal gyrus, left middle occipital gyrus, right postcentral gyrus, and right inferior parietal lobule (Table 4 and Figure 3c, d).

**Table 2:** Demographic and clinical features of the responder and poor responder groups

	Responders	Poor responders
Number	16	13
Age, years	45.3 ± 9.1	39.5 ± 12.6
Duration of illness, years	3.0 ± 1.9	3.2 ± 2.4
Tender points, 0 to 18	13.9 ± 2.0	15.4 ± 2.8
VAS at baseline, 0 to 100	81.3 ± 9.6	86.9 ± 11.8
VAS after gabapentin, 0 to 100	15.6 ± 7.3	83.1 ± 16.0
Dose of gabapentin, milligrams	2,156.3 ± 427.4	2,215.4 ± 450.6

VAS, visual analogue scale.

**Table 3:** Regions of significant hyperperfusion and hypoperfusion in the responder group

	κ	Z score	x, mm	y, mm	z, mm	Localization
Hyperperfusion	419	4.82	-24	-4	68	Left superior frontal gyrus
	169	3.80	-30	-42	68	Left postcentral gyrus
Hypoperfusion	420	4.23	2	60	-16	Right medial frontal gyrus
	2,053	4.66	-44	-4	10	Left insula
	420	4.23	-18	30	-24	Left inferior frontal gyrus
	258	4.38	-14	-32	-14	Left culmen

Results are listed by clusters. κ value, Z score, Talairach coordinates of peak voxel, and anatomic localization are provided for each cluster.

**Table 4:** Regions of significant hyperperfusion and hypoperfusion in the poor responder group

	κ	Z score	x, mm	y, mm	z, mm	Localization
Hyperperfusion	253	4.87	66	-10	34	Left precentral gyrus
	758	4.55	2	-62	14	Right posterior cingulate
	3,168	4.16	40	-76	34	Right precuneus
	710	3.98	-20	-8	66	Left middle frontal gyrus
	1,052	3.95	54	-48	8	Right superior temporal gyrus
	820	3.57	-38	-88	-2	Right middle occipital gyrus
	201	3.52	10	-36	82	Left postcentral gyrus
	134	3.54	48	-42	58	Left inferior parietal lobule
Hypoperfusion	207	4.38	-14	36	-26	Left orbital gyrus

Results are listed by clusters. κ value, Z score, Talairach coordinates of peak voxel, and anatomic localization are provided for each cluster.

**Figure 4:**
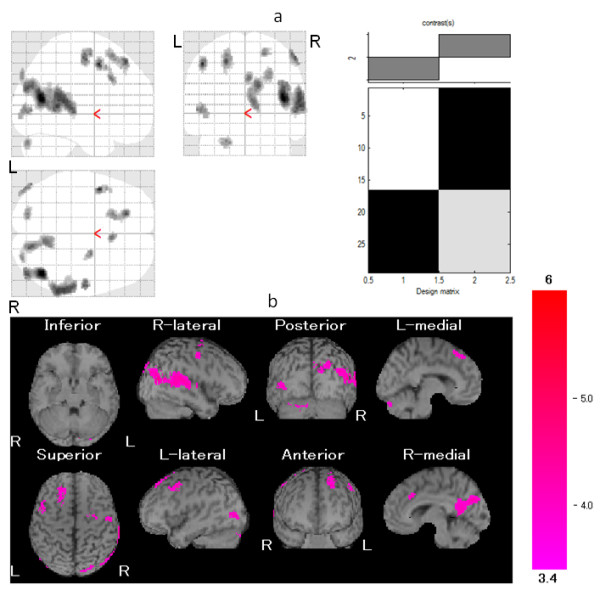
**Comparison of regional cerebral blood flow (rCBF) between the responder group and the poor responder group**. Maximum intensity projections of Statistic Parametric Mapping 2 (SPM2) results from comparison of rCBF between the responder and poor responder groups are shown. **(a, b) **The poor responder group exhibited hyperperfusion in the right middle temporal gyrus, left middle frontal gyrus, left superior frontal gyrus, right postcentral gyrus, right precuneus, right cingulate, left middle occipital lobule, and left declive. Height threshold is less than 0.001, corrected for multiple comparison.

Furthermore, compared with responders, poor responders exhibited hyperperfusion in the right middle temporal gyrus, left middle frontal gyrus, left superior frontal gyrus, right postcentral gyrus, right precuneus, right cingulate, left middle occipital gyrus, and left declive in the cerebellum (Table 5 and Figure 4a, b). There was no area with significant hypoperfusion in the poor responder group compared with the responder group. The sensitivity, specificity, positive predictive value, and negative predictive value for prediction of poor response to gabapentin are shown in Table 6. The following high positive likelihood ratios (greater than 10) were observed: right middle temporal gyrus, 13.6; left superior frontal gyrus, 8 (specificity 100%); right precuneus, 8 (specificity 100%); left middle occipital gyrus, 12.4; and left declive, 12.4.

**Table 5:** Regions of significant hyperperfusion in the poor responder group compared with the responder group

	κ	Z score	x, mm	y, mm	z, mm	Localization
Hyperperfusion	1,260	4.08	42	-62	16	Right middle temporal gyrus
	95	3.88	-46	6	50	Left middle frontal gyrus
	95	3.88	-20	38	52	Left superior frontal gyrus
	69	3.67	56	-12	56	Right postcentral gyrus
	578	3.67	14	-76	28	Right precuneus
	59	3.58	4	20	36	Right cingulate
	70	3.54	-20	-80	4	Left middle occipital lobule
	77	3.51	-20	-80	-26	Left declive

Results are listed by clusters. κ value, Z score, Talairach coordinates of peak voxel, and anatomic localization are provided for each cluster.

**Table 6:** Sensitivity, specificity, and positive and negative predictive values based on pretreatment regional cerebral blood flow of each cluster

Localization	Sensitivity, %	Specificity, %	PPV, %	NPV, %
Right middle temporal gyrus	84.6	93.8	91.7	88.2
Left middle frontal gyrus	84.6	81.3	78.6	86.7
Left superior frontal gyrus	69.2	100	100	80
Right postcentral gyrus	84.6	68.8	68.8	84.6
Right precuneus	76.9	100	100	84.2
Right cingulate	76.9	81.3	76.9	81.3
Left middle occipital lobule	76.9	93.8	90.9	83.3
Left declive	76.9	93.8	90.9	83.3

NPV, negative predictive value; PPV, positive predictive value.

## Discussion

### Brain perfusion in fibromyalgia patients

In the present study, performed without noxious stimuli in patients with FM, we found significant hyperperfusion in the middle frontal gyrus, medial frontal gyrus, precuneus, cuneus, middle temporal gyrus, postcentral gyrus, and inferior parietal lobule and significant hypoperfusion in the culmen. Although several SPECT studies revealed abnormal rCBF in patients with FM, the findings were inconsistent, as noted in the Introduction, and also differed from those of the present study. Differences in experimental conditions could explain these discrepancies in findings. First, Mountz and colleagues [3] and Kwiatek and colleagues [4] used ^99m^Tc-HMPAO rather than ^99m^Tc-ECD as a tracer. Although uptakes of ^99m^Tc-ECD and ^99m^Tc-HMPAO in the brain exhibit a fixed distribution, these tracers have different pharmacokinetics and yield different image qualities. ^99m^Tc-ECD uptake is known to reflect cerebral blood flow more closely than ^99m^Tc-HMPAO and to be more sensitive in estimating regional metabolic rate, especially in the medial temporal lobe of the cortex. Second, although Guedj and colleagues [5] used ^99m^Tc-ECD (as in the present study), their subjects' characteristics differed from ours. Subjects in the study of Guedj and colleagues were hospitalized because of treatment failure, whereas our subjects were CNS drug-naive outpatients without major depressive disorder. Our findings were therefore not affected by CNS drugs. In contrast, subjects in the study of Guedj and colleagues may have been affected by administered CNS drugs since their hospitalization was due to treatment failure. Our findings for FM patients without previous use of CNS drugs may have reflected primary changes in rCBF in FM.

Interestingly, the regions of hyperperfusion observed in the present study have been previously referred to as the 'default mode network' (DMN) [9,10]. Previous functional magnetic resonance imaging (fMRI) studies showed that DMN exhibited decreased neural activity during goal-related tasks compared with that at rest [11,12] and that it appears to be involved in various aspects of self-referential processing [13], which in concerted action maintain the brain resting state [14]. In other words, in the normal brain, the DMN provides 'a balance of opposing forces' to enhance 'the maintenance of information for interpreting, responding to, and even predicting environmental demands' ([12]. Although the mechanism by which it does so is unclear, pain is known to interact with the DMN. Acute pain [15] is known to induce deactivation in DMN regions, whereas chronic pain may be associated with disruption of the DMN [16]. Furthermore, perception of somatosensory stimuli near sensory threshold is facilitated by decreased DMN activity in a brief pre-event resting period [9]. Also, in patients with FM, continuous perception of pain may alter the brain resting state. Since our findings did not include connectivity data, we are unable to address the question of the relationship between the pathophysiology of FM and DMN. However, the present study yielded the first observation of hyperperfusion in DMN regions in patients with FM.

### Differences in brain perfusion between responders to gabapentin and non-responders among fibromyalgia patients

Gabapentin is thought to exert antinociceptive effects primarily by modulation of calcium channels via α_2_δ binding, which reduces the release of several neurotransmitters involved in pain processing, such as glutamate, noradrenaline, and substance P [17]. However, the mechanism of action of gabapentin in the human brain has yet to be elucidated. In this study, pronounced pretreatment hyperperfusion in the right middle temporal gyrus, left superior frontal gyrus, right precuneus, left middle occipital gyrus, and left declive appeared to be predictive of poor response to gabapentin given the high positive likelihood ratios obtained for these regions. These findings are not consistent with a previous finding for non-responders to ketamine, in which hypoperfusion in the medial frontal area was observed [18]. As noted above, subjects in the study of Guedj and colleagues were hospitalized because of treatment failure whereas our subjects were CNS drug-naive outpatients without major depressive disorder, and our findings were correspondingly not affected by CNS drugs. In contrast, subjects in the study of Guedj and colleagues may have been affected by previously given CNS drugs because their hospitalization was due to treatment failure. Interestingly, areas predictive of poor response to gabapentin are involved in the DMN. It thus appears possible that FM, especially intractable FM with gabapentin, is related to DMN functioning.

Several limitations of this study should be considered. First, as we have no post-treatment SPECT data, further work will be needed to clarify the potential reversibility of our findings after treatment. Second, this was an open study with a flexible dose design, which limited assessment of the effectiveness of gabapentin. Third, because the trial was only 12 weeks in duration, its results may not be generalizable to longer treatment periods, and the long-term efficacy of gabapentin should be explored in future clinical trials. Fourth, the results of this trial may not be generalizable to patients with certain comorbid psychiatric disorders or patients with comorbid rheumatologic or other medical disorders since patients with these conditions were excluded from the trial. Finally, other brain imaging studies, such as positron emission tomography and fMRI, will be needed to clarify the pathophysiology of FM and the characteristics of responders to gabapentin treatment.

## Conclusions

The present study showed significant hyperperfusion in areas associated with the DMN in addition to abnormalities in the sensory dimension of pain processing and affective-attentional areas in patients with FM. Furthermore, hyperperfusion in these areas was highly predictive of poor response to gabapentin. Further studies yielding fMRI and connectivity data will be needed to clarify the association between pathogenesis of FM and DMN.

## Abbreviations

CNS: central nervous system; DMN: default mode network; ECD: ethyl cysteinate dimer; FM: fibromyalgia; fMRI: functional magnetic resonance imaging; HMPAO: hexamethylpropylene amine oxime; MNI: Montreal Neurological Institute; rCBF: regional cerebral blood flow; SPECT: single-photon emission computed tomography; SPM: Statistic Parametric Mapping; Tc: technetium; TP: tender point; VAS: visual analogue scale.

## Competing interests

The authors declare that they have no competing interests.

## Authors' contributions

CU conceived the hypothesis for the study, participated in data collection, conducted the initial statistical analyses, wrote the first draft of the manuscript, and was primarily responsible for the process of manuscript writing. KH, ND, AN, and HN contributed to data management and statistical analyses. KN and HA participated in study design and analysis and interpretation of data. All authors critically reviewed, contributed to, and approved the final manuscript.
